# Next-generation sequencing shows the genomic features of ovarian clear cell cancer and compares the genetic architectures of high-grade serous ovarian cancer and clear cell carcinoma in ovarian and endometrial tissues

**DOI:** 10.7717/peerj.14653

**Published:** 2023-01-26

**Authors:** Meifu Gan, Zaixian Tai, Yijian Yu, Chao Zhang, Juan Xu

**Affiliations:** 1 Department of Pathology, Taizhou Hospital affiliated to Wenzhou Medical University, Linhai, Zhejiang Province, China; 2 Geneplus Shenzhen, Shenzhen, Guangdong province, China

**Keywords:** OCCC, HGSOC, ECCC, Genetic architectures, Genomic features

## Abstract

Ovarian clear cell carcinoma (OCCC) is a special histological type of epithelial ovarian cancer (EOC) that is not derived from epithelial cells of the ovarian or fallopian tube as the most common type of ovarian cancer, high-grade serous ovarian carcinoma (HGSOC), but is closely related to endometriosis and similar to endometrial clear cell carcinoma (ECCC) at morphologic and phenotypic features. However, limited data was shown in OCCC genomic features and compared with that in OCCC, HGSOC and ECCC. Herein, we utilized next-generation sequencing analysis of a panel of 1,021 genes to profile the mutational alterations in 34 OCCC and compared them to those from HGSOC (402 cases) and ECCC (30 cases). In result, the ARID1A and PIK3CA are high-frequency mutations of OCCC. Clonal architectures showed that all the mutations of genes occur in the later stage in the OCCC progress, whereas KRAS mutation is the earlier event compared with mutation of ARID1A or PIK3CA, which usually occurs in a group of ARID1A or PIK3CA mutations. The mutation frequency of main driver genes is similar between OCCC and ECCC, while TP53 is the main mutation in HGSOC and ECCC. Shared mutational signatures between OCCC and ECCC tissues with commonly observed a C>T change indicated a common carcinogens-exposed between these two carcinomas, but HGSOC and ECCC have common and distinct mutational signatures across cohorts respectively. In addition, we identified some novel CNV gains in NF1, ASXL1, TCF7L2, CREBBP and LRP1B and loss in ATM, FANCM, RB1 and FLT in OCCC. Our study offered a new perspective for OCCC tumorigenesis from two organs, the ovary and uterus, at genomic architectures and revealed novel CNV events for helping to provide theoretical support for OCCC treatment.

## Introduction

Ovarian clear cell carcinoma (OCCC) is a specific histological type of ovarian malignant tumor (WHO, 2020), accounting for 5% to 25% of epithelial ovarian cancer (EOC), especially in Asians, it has a rising and younger trend year by year (Anglesio et al., 2011; Machida et al., 2019; Pozzati et al., 2018). Unlike high-grade serous ovarian cancer (HGSOC), OCCC is not originated from epithelial cells of the ovarian or fallopian tube but is closely related to endometriosis (Gadducci, Lanfredini & Tana, 2014; Jenison et al., 1989; Munksgaard & Blaakaer, 2012). Moreover, its morphologic and phenotypic features are more similar to those in endometrial clear cell carcinoma (ECCC) (Ju et al., 2018; Travaglino et al., 2020). Although previous work tried to reveal the tumorigenic mechanism of OCCC at the genomic and molecular level, the large contents remain obscure.

High-frequency somatic mutations in OCCC include AT-rich interaction domain 1A (ARID1A) and phosphatidylinositol-4,5-bisphosphate3-kinase (PIK3) catalytic subunit alpha (PIK3CA), that is similar in endometriosis without cancer, while the common mutation in HGSOC and ECCC is tumor protein 53 (TP53) (Anglesio et al., 2017; Baniak et al., 2019; Wiegand et al., 2010). To date, there are no approved specific targeted therapies for OCCC. Patients with OCCC currently received the same chemotherapy regimen as HGSOC: cisplatin-based combination chemotherapy. However, the effective rate of cisplatin in the treatment of OCCC is only 11%–50%, and the majority of patients with OCCC are resistant to cisplatin (Crotzer et al., 2007; Takano, Tsuda & Sugiyama, 2012). The survival rate of patients with OCCC in the advanced stage is much lower than other EOC subtypes (Sugiyama et al., 2000). Therefore, to improve the prognosis of patients with OCCC, it is necessary to strengthen the research on the pathogenesis of OCCC and develop a more effective therapeutic strategy for OCCC.

Herein, we detected the mutational features of 68 tissue samples (tumor and matched normal tissue) from 34 patients with OCCC by using a 1,021-gene panel of next-generation sequencing, further integrated sequencing data (MSK panel) of 30 patients with ECCC and whole-exome sequencing (WES) data of 402 patients with HGSOC from public databases of The Cancer Genome Atlas (TCGA), exhibiting common and unique genetic alteration, including clonal architecture, mutation signatures and copy number variations (CNV) in OCCC, HGSOC, and ECCC to illuminate the underlying mechanisms of OCCC tumorigenesis and progress.

## Material and Methods

### Sample collection

The ECCC sequencing data (MSK panel) were downloaded from the literature (DeLair et al., 2017) and the HGSOC sequencing data were downloaded from the cBioportal database (http://www.cbioportal.org/). Moreover, the OCCC data (1,021 panel) were collected from the Geneplus genomic data bank from May 2015 to August 2022. The detailed clinico-pathological and sample information were shown in Table S1. The retrospective study was designed and conducted in accordance with the Declaration of Helsinki. Written informed consent was granted in sample collection, gene sequencing, and data analysis, with the obtained information authorized for publishing. It was approved by the Institutional Review Board (IRB) of Taizhou Hospital of Zhejiang Province (No. K20220844).

### Targeted capture sequencing

A tissue kit was used to extract genomic DNA (gDNA) from OCCC samples and matched normal tissues (Qiagen, Hilden, Germany). Following the manufacturer’s instructions, sequencing libraries were created using the KAPA DNA Library Preparation Kit (Kapa Biosystems, MA, USA). For both biopsy samples, barcoded libraries were hybridized to a panel of 1,021 genes with full exons, chosen introns from 288 genes, and selected regions from 733 genes. The comprehensive gene list was described in our earlier research (Wang et al., 2020). The DNBSEQ-T7RS (BGI, Shenzhen, China) with 100 bp paired-end reads was used to sequence the DNA.

### Mutation, somatic interactions, and somatic copy number variation calling

MuTect was utilized to identify minor insertions and deletions (Indels), as well as single nucleotide variations (SNVs) (version 1.1.4). For quality control, somatic mutations were only found if they met the following criteria: (i) they were present in less than 1% of the population in the 1000 Genomes Project, the Exome Aggregation Consortium, and the Genome Aggregation Database (gnomAD) (https://gnomad.broadinstitute.org); (ii) they weren’t present in paired germline DNA from normal tissues; and (iii) they were found in at least five high-quality reads and without paired-end reads bias. The maftools was used to delineate the mutational landscape (Mayakonda et al., 2018).

The somatic interactions landscape was drawn by maftools (Mayakonda et al., 2018) to identify gene sets mutated in a mutually exclusive or co-occurring manner. A Fisher’s exact test on a 2*2 contingency table including frequencies of mutant and nonmutated samples is used to assess the pattern of exclusivity or co-occurrence for a pair of genes.

Focal somatic copy number variation (SCNV) was identified by CONTRA (Li et al., 2012) and the frequency of larger fragmental CNV was calculated. To compare the mutational frequency across each tumor subtype, the overlapped region was obtained from each panel. The top10 CNV genes were extracted from the HGSOC segment file and OCCC 1021 panel results.

### Analysis of mutational signature and clonal architecture

The mutational signature analysis was performed with unfiltered somatic mutations using the R package YAPSA (Hubschmann et al., 2021) and matched to the COSMIC signature database (https://cancer.sanger.ac.uk/cosmic/signatures, Mutational Signatures V3.3).

The variant allele frequency (VAF) ratio of mutations was used to infer the clonality of mutational events (Gerlinger et al., 2012). Specifically, the VAF ratio was calculated by dividing the VAF of each mutation by the maximum VAF observed in the same sample. A higher VAF ratio suggested the respective event occurred at an earlier stage during tumor progression. Mutations with VAF ratios >0.6 were determined as clonal mutations while the rest were considered subclonal mutations.

### Statistics

Two-sided Mann–Whitney and Fisher’s exact tests were performed on GraphPad Prism (version 8.02) or R (version 3.6.1) to generate the *P* value. For all tests, a *P* value <0.05 was considered statistically significant.

## Results

### Somatic mutations and indels in OCCC, HGSOC, and ECCC

**Figure 1:**
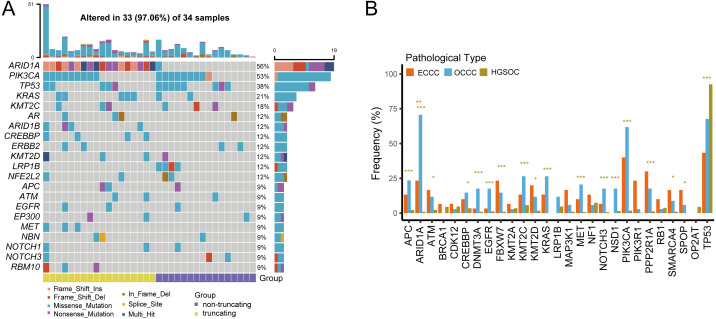
The mutational landscape of OCCC and the difference of high frequency driver mutations in OCCC, HGSOC and ECCC. (A) Mutations with high mutational frequencies in OCCC. (B) The difference of high frequency driver mutations in OCCC, HGSOC and ECCC. Claybank asterisk (*) indicates the significant difference between OCCC and HGSOC, orange asterisk (*) indicate the significant difference between OCCC and ECCC, *p < 0.05, **p < 0.01, ***p < 0.001. OCCC, clear cell carcinoma of the ovary; HGSOC, high-grade serous ovarian carcinoma; ECCC, endometrial clear cell carcinoma.

We examined the mutational burdens in 34 samples with OCCC and observed 538 somatic single nucleotide variations (SNVs) and small insertions and deletions (Indels) in the tumor samples. In OCCC, mutations of top10 genes were all relatively high (12%–56%), the main mutations were ARID1A (56%) and PIK3CA (53%), following TP53 (38%), KRAS (21%), KMT2C (18%). Low-density lipoprotein receptor-related protein 1B (LRP1B) mutation occurred only in samples without ARID1A truncating mutations in OCCC, while the ARID1B/CREBBP/ MET/RBM10 mutation occurred in the ARID1A in truncating group (Fig. 1A). After taking the intersection of the OCCC sequencing panel, 1,653 and 750 mutations were identified in HGSOC and ECCC cohorts respectively. According to a prior study, HGSOC had the highest TP53 mutation frequency (92%) and the low mutation rates for other genes (NF1, KMT2C, CDK12, LRP1B, and TOP2A were the only mutations greater than or equal to 5%) (Fig. S1A). In the ECCC cohort, the genes TP53 (43%), PIK3CA (40%), PPP2R1A (30%), ARID1A (23%), PIK3R1 (23%), FBXW7 (23%), and KMT2D (20%) had high-frequency mutations (Fig. S1B). We then compared the frequencies of mutations (top 10 genes) in known cancer-associated genes in OCCC, HGSOC, and ECCC. In result, we observed similar mutational frequencies in some canonical cancer driver genes between OCCC and ECCC cohorts, such as PIK3CA (OCCC 53% *vs.* ECCC 40%), TP53 (OCCC 38% *vs.* ECCC 43%), KRAS (OCCC 21% *vs.* ECCC 13%), APC (OCCC 9% *vs.* ECCC 13%), and KMT2C (OCCC 18% *vs.* ECCC 13%), while only the mutation rate of ARID1A in OCCC was significantly higher than that in ECCC (*p* < 0.01; Fig. 1B). In addition, only the mutation rate of TP53 was significantly higher in HGSOC than that in OCCC, while 17/27 the mutation rates of genes in OCCC were significantly higher than that in HGSOC (*p* < 0.001; Fig. 1B).

**Figure 2:**
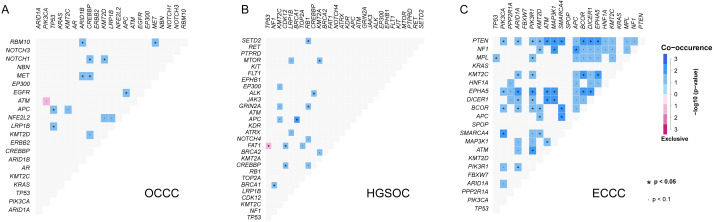
The analysis of mutation gene interaction in OCCC, HGSOC and ECCC. (A) The analysis of mutation gene interaction in OCCC. (B) The analysis of mutation gene interaction in HGSOC. (C) The analysis of mutation gene interaction in ECCC. OCCC, clear cell carcinoma of the ovary; HGSOC, high-grade serous ovarian carcinoma; ECCC, endometrial clear cell carcinoma. *p < 0.05. *p* < 0.1.

Further gene interaction analysis showed that DNMT3A (not shown in top 10 genes) and PIK3CA were mutually exclusive occurrences, APC and TP53/KMT2C/ACIN1/FBXW7/EGFR were co-occurrence relationships in OCCC samples (*p* < 0.05; Fig. 2A). In HGSOC, TP53 and FAT1 were mutually exclusive occurrence relationships, APC co-occurred with KMT2C/BRAC1, and MTOR co-occurred with KMT2A (*p* < 0.05, Fig. 2B). In ECCC, ARID1A co-occurred with PTEN/ KMT2C/ EPHA5/DICER1/ECOR/PI3KR1, and TP53 co-occurred with MEL, and PIK3CA co-occurred with PTEN/ EPHA5/BCOR/SMARCA4 (*p* < 0.05, Fig. 2C). These results suggested that there were differences in the nature of interacting oncogenes in these three tumor types.

### Clonal architectures in OCCC, HGSOC, and ECCC

**Figure 3:**
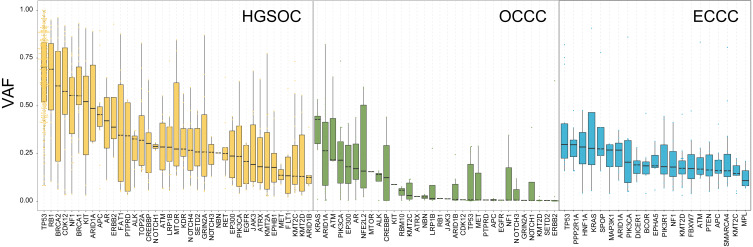
The exhibition of timing order of mutational genes in OCCC, HGSOC and ECCC. The distributions of VAF value of high frequency driver mutations in HGSOC, OCCC and ECCC. VAF assessment was performed as described in “Materials and Methods”. OCCC, clear cell carcinoma of the ovary; HGSOC, high-grade serous ovarian carcinoma; ECCC, endometrial clear cell carcinoma. VAF variant allele frequency.

To explore the probable timing order of the mutation events arose in OCCC, HGSOC, and ECCC, we gauged the variant allele frequency (VAF) ratio of somatic mutations and Indels in these tumors with the method mentioned in previous literature (Gerlinger et al., 2012). The median of high/low VAF represents an early or late event, respectively. As shown in Fig. 3, driver genes like TP53 and RB1 were determined as clonal mutations with high VAF ratios (>0.6), indicating they might occur at an earlier stage compared with other subclonal mutations with low VAF ratios (<0.5), and might play crucial roles in HGSOC tumorigenesis. By contrast, the median VAF value of all genes was less than 0.5 in OCCC and ECCC, suggesting that all alterations were the later events in these two malignancies as subclonal mutations. Although the mutational frequency of ARID1A and PIK3CA were higher than other genes, the median VAF value of KRAS was highest and the TP53 mutation arose later in OCCC. In ECCC, the median VAF values of TP53, ARID1A, KRAS and PIK3CA mutation were low as later events. These results suggested that the driver genes triggered cancer in OCCC, ECCC, and HGSOC were different, and different mutations of genes were presented in these tumors at different stages, some mutations were newly obtained during OCCC and ECCC malignant evolution.

### Mutational signatures in OCCC, HGSOC, and ECCC

**Figure 4:**
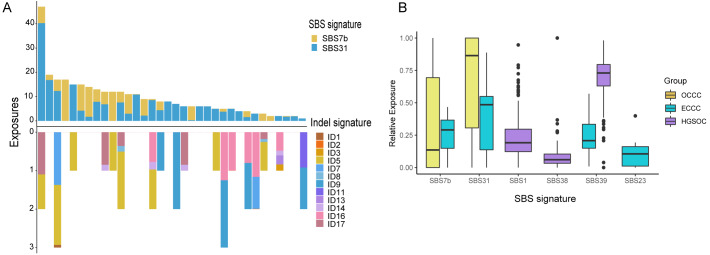
Exposing Contribution of COSMIC signatures in OCCC patients and the distribution of mutational signatures in OCCC, HGSOC and ECCC. (A) Panel at the top, exposing contribution of SBS signatures; panel in the middle, OCCC patients in our cohort (*N* = 34); panel at the bottom, exposing contribution of Indel signatures. (B) Boxplot shows shared, OCCC-specific, HGSOC-specific, and ECCC-specific mutational proportion per signature. OCCC, clear cell carcinoma of the ovary; HGSOC, high-grade serous ovarian carcinoma; ECCC, endometrial clear cell carcinoma.

The mutational signatures can reflect the potential carcinogens exposed in tumorigenesis and progress. We identified somatic SNVs and Indels corresponding Catalogue of Somatic Mutations in Cancer (COSMIC) single base signatures (SBS) and insertion and deletion signatures (ID) in OCCC. Somatic SNVs were mainly attributed to two SBSs in the COSMIC database, SBS7b, and SBS31 specifically, while Indels were mainly attributed to ID5, ID11, ID16, and ID17 (Fig. 4A). We then identified the SNVs mutational signatures patterns of OCCC, HGSOC, and ECCC to gain insight into the different etiology of those tumors. As shown in Fig. 4B, mutation signatures of OCCC were shared with ECCC, including SBS31 and SBS7b. C >T (cytosine>thymine transitions) mutational pattern of SBS7b was frequently found in cancers of the skin and associated with exposure to ultraviolet light (Alexandrov et al., 2020). SBS31, characterized by C >T mutations, may be due to platinum drug treatment. The proposed etiology of ECCC-specific SBS23 of the C >T pattern is still unknown. SBS39, featured with predominant C >G alterations, was shared in HGSOC and ECCC. It was found with high distribution in head and squamous cell carcinoma, breast cancer, and ovary-adenoma and without known proposed etiology (Alexandrov et al., 2020). SBS1 and SBS38 were private signatures in HGSOC. C >T change of SBS1 is an endogenous mutational process initiated by spontaneous or enzymatic deamination of 5-methylcytosine to thymine which generates G: T mismatches in double-strand DNA, which is considered as a cell division/mitotic clock. SBS38, a pattern of C>A mutation, was found only in melanomas with potential indirect damage from ultraviolet light. These results suggested that the potential mutational signature patterns in OCCC were more similar to ECCC than that in HGSOC.

### Somatic copy number variation in OCCC, HGSOC, and ECCC

**Figure 5:**
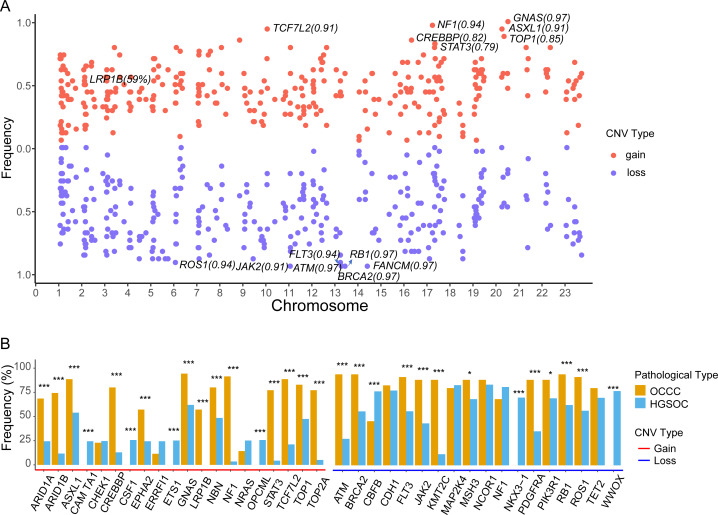
The identify of copy number variations in OCCC and the comparing of the main CNV in genes between OCCC and HGSOC. (A) The identify of focal copy number variations in OCCC. (B) The different of CNV events (TOP10) between OCCC and HGSOC. OCCC, clear cell carcinoma of the ovary; HGSOC, high-grade serous ovarian carcinoma; CNV, copy number variation; Chr, chromosomes.

The focal somatic copy number variation (CNV) amplification and deletion were extracted from OCCC 1021 panel by CONTRA (Li et al., 2012). The results as shown in Fig. 5A and Table S2, we exhibited the frequencies of gain or loss of CNVs located on chromosomes (Chr) and focus the high frequency of CNV gain events in genes such as GNAS (0.97) on Chr20q13.32, NF1 (0.94) on Chr17q11.2, ASXL1 (0.91) on Chr20q11.21, TCF7L2 (0.91) on Chr10q25.2, CREBBP (0.81) on chr16p13.3, LRP1B (0.59) on 2q22.2 and loss events in ATM (0.97) on 11q22.3, BRCA2 (0.97) on 13q13.1, FANCM (0.97) on 14q21.2, RB1 (0.97) on 13q14.2, FLT3 (0.94) on 13q12.2, ROS1 (0.94) on 6q22.1 and JAK2 (0.91) on 9p24.1. Further comparing the top10 genes with a high frequency of CNV amplification or deletion between OCCC and HGSOC, we obtained significantly different cancer-associated genes at the CNV level in these two subtypes (Fig. 5B). In result, canonical oncogenes such as ARID1B, ARID1A1, ASXL1, CREBBP, EPHA2, GNAS, NBN, LRP1B, NF1, STAT3, TCF7L2, TOP1, TOP2A, KMT2C were gains in OCCC, whereas CAMTA1, CSF1, ETS1, OPCML were gained in HGSOC (*p* < 0.001, Fig. 5B). Tumor suppressor genes such as NKX3 −1, CBFB and WWOX were loss in HGSOC and AMT, BRCA2, FLT3, JAK2, KMT2C, PDGFRA, PIK3R1, RB1, ROS1 were loss in OCCC (*p* < 0.001, Fig. 5B).

## Discussion

In our study, we identified firstly the genomic features of OCCC, then distinguished the similarities and differences of the genetic architectures and clonal patterns in OCCC, ECCC, and HGSOC. In result, we elaborated on the similarity of mutation rate of key driver gene, mutational signature, and clonal architectures between OCCC and ECCC, and different genetic features between OCCC and HGSOC. Importantly, our data emphasized KRAS potential role in samples with ARID1A or PIK3CA mutation in OCCC, identified LRP1B mutation group only occurred in samples with non-ARID1A truncation mutation, distinguished the potential carcinogens-exposed of those tumors by mutational signatures and unearthed several novel drivers in CNV level that might essentially contribute to OCCC tumor progression.

The frequent mutation genes in OCCC are PIK3CA (Kato et al., 2019; Kuo et al., 2009) and ARID1A (Katagiri et al., 2012; Wiegand et al., 2010), which are frequently co-occurrence (Oliveira et al., 2021). In our results, the coexistence of PIK3CA and ARID1A mutations accounted for 29% (10/34) of all OCCC samples, while mutation rates of TP53 and KRAS are 38% and 21%, respectively. We found that LRP1B was found only in samples without ARID1A truncating mutations in OCCC. As a tumor suppressor, LRP1B mutation was associated with favorable outcomes to immune checkpoint inhibitor across multiple cancer types (Brown et al., 2021), and LRP1B protein was a predictor of response to pegylated liposomal doxorubicin in patients with ovary cancer (Dionisio de Sousa et al., 2021). However, the ARID1B/CREBBP/MET/RBM10 mutation only occurred in the samples with ARID1A truncating mutation which is related to ARID1A absence or low expression. Jung et al. (2021) reported that low ARID1A expression correlated with poor overall survival of OCCC patients (1011 cases) by a meta-analysis. These results provided an experimental basis for the treatment choice of OCCC.

In Fig. 1B, TP53 is the main mutation and associated with DNA damages in HGSOC and ECCC (Baniak et al., 2019; Kroeger Jr & Drapkin, 2017; Vang et al., 2016). The histological and molecular phenotypes are similar in the expression of TP53, ER, PR, HIF1 *β* and napsinA between OCCC and ECCC (Ju et al., 2018; Lim et al., 2015), while mutation frequencies of ARID1A in our OCCC cohort were observed significantly higher than in ECCC cohort, and mutational frequencies in some canonical cancer driver genes are similar between these two cohorts, such as PIK3CA, TP53, KRAS, APC, and KMT2C, indicating that they might play pivotal roles in clear cell carcinoma tumorigenesis. In addition, we found a mutually exclusive relationship between DNMT3A and PIK3CA in OCCC. TP53 and FAT1 were mutually exclusive in HGSOC (Fig. 2). The mutually exclusive relationship in genes is associated with tumor types (Cisowski & Bergo, 2017). These results indicated that although there was a similar genetic underpinning between OCCC and ECCC, the major gene mutation was ARID1A in OCCC, while in ECCC and HGSOC, the dominant gene mutation was TP53, which may be associated with organ selection (ovary or uterus) for tumorigenesis.

ARID1A did not lead to tumor formation by itself, and the coexistent ARID1A-PIK3CA mutations promote OCCC tumor formation (Chandler et al., 2015; Yamamoto et al., 2012). In our result, all mutations of genes were considered as later events in OCCC tumorigenesis due to the subclone architecture by analyzing the VAF values of mutations in OCCC. Although the mutation frequencies of ARID1A and PIK3CA are the highest, the VAF value of KRAS mutation is the highest, indicating that KRAS mutation is the earlier event compared with ARID1A and PIK3CA in OCCC tumorigenesis. Notably, the KRAS mutation occurs only in the group of ARID1A or PIK3CA mutations, implying that KRAS mutation might play a crucial role in ARID1A or PIK3CA triggered OCCC as an early driver mutation. In the HGSOC, the clonal mutation genes were TP53 (Vang et al., 2016) and RB1, which occurred in an earlier stage and initiated the HGSOC tumorigenesis, whereas the VAF value of TP53 mutation was less than 0.25 in OCCC, indicating a TP53 mutation is a later event in OCCC progress. The VAF value of mutations in ECCC was low like that in OCCC, the timing order of the mutation events arose in the tumor was different, and mutations such as TP53, PPP2R1A, HIF1A, KRAS, SPOP, MAP3K1, and PIK3CA are almost concurrent by the similar VAF value of mutation. These results suggested that based on the prodromal disease endometriosis, malignant changes were caused by mutations acquired in the later stages of OCCC, which is similar to ECCC and not completely different from HGSOC at gene clonal architecture.

SBS7b and SBS31 were characterized by C >T mutations in OCCC (Fig. 4A), are also shared SBSs in OCCC and ECCC (Fig. 4B), which was associated with exposure to ultraviolet light (Alexandrov et al., 2020) and cannot dismiss that the observed signature might be partially due to the patients with platinum drug treatment. ID5 was characterized by predominant single T base deletion, which was observed particularly predominant in OCCC (Fig. 4A), and was reported to be a clock-like signature associated with patients’ ages (Alexandrov et al., 2020). Maru et al. (2017) reported that the mutation of the C to T transition was the most frequently observed in OCCC, but not demonstrated the potential carcinogens of OCCC carcinogenesis corresponding to this alternation. SBS39 featured with predominant C >G alterations, was shared in HGSOC and ECCC, which was also found with high distribution in head and squamous cell carcinoma, breast cancer, and ovary-adenoma and without known proposed etiology (Alexandrov et al., 2020), while the proposed etiology of ECCC-specific SBS23 of C >T pattern is still unknown. The potential carcinogens-exposed of HGSOC were associated with cell division/mitosis and potential indirect damage from ultraviolet light by analyzing private signatures of HGSOC, SBS1 and SBS38. Our findings and previous reports indicated that the potential mutational signatures in OCCC were more similar to ECCC compared with HGSOC. The discrepancy in signature distribution is considered reasonable given that OCCC and ECCC co-occur from the endometrium.

Currently, only a few studies have reported CNV events in OCCC. The copy number gains in ERBB2, HNF1beta, STAT3, GNAS, MYC, PIK3CA, KRAS, CCNE1, IL6/ IL6R and loss in TET2, TSC1, BRCA2 and SMAD4 in OCCC (Kim et al., 2018; Murakami et al., 2017). Among CNV events, we detected focal CNV events in genes in OCCC and identified the novel gains in NF1, ASXL1, TCF7L2, CREBBP, and LRP1B and loss in ATM, FANCM, RB1, and FLT (Fig. 5A and Table S2). Then, we further explored the different CNV events between OCCC and HGSOC, OCCC CNV gains in ARID1B, ARID1A1, ASXL1, CREBBP, EPHA2, GNAS, NBN, LRP1B, NF1, STAT3, TCF7L2, TOP1, TOP2A, and KMT2C and loss in AMT, BRCA2, FLT3, JAK2, KMT2C, PDGFRA, PIK3R1, RB1, and ROS1 (Fig. 5B). Among OCCC CNV gains events, ARID1B, ARID1A1, and ASXL1 are associated with chromatin remodeling and modification. In novel identified CNV events of OCCC, CREBBP is involved in tumorigenesis in various cancers, and knockdown CREBBP can promote chemo-sensitivity in ovarian cancer cells (Hu et al., 2021), while EPHA2 is associated with platinum-resistant in HGSOC, and RSK inhibitors effectively sensitized cells with EphA2^high^ to the therapy-induced apoptosis (Moyano-Galceran et al., 2020), LRP1B can act as a predictor of response to pegylated liposomal doxorubicin for ovary cancer (Dionisio de Sousa et al., 2021). These results provided more potential targets for OCCC treatment.

## Conclusions

In summary, our study is a new attempt to compare simultaneously genetic features of OCCC to carcinoma of the uterus or ovaries to further dissected the mechanisms that might essentially contribute to OCCC tumorigenesis. Our data exhibited similar genomic features between OCCC and ECCC, and OCCC is significantly different from HGSOC at the genomic level. Importantly, we emphasized the role of key gene mutation in OCCC tumorigenesis and revealed clonal architecture and novel CNV events of OCCC. Our results provided a new experimental basis for therapeutic targets of OCCC.

##  Supplemental Information

10.7717/peerj.14653/supp-1 Figure S1 The mutational landscape of HGSOC and ECCC (A) Mutations with high mutational frequencies in HGSOC. (B) Mutations with high mutational frequencies in HGSOC. ECCC, endometrial clear cell carcinoma; HGSOC, high-grade serous ovarian carcinoma.

10.7717/peerj.14653/supp-2 Table S1 Supplementary Table1

10.7717/peerj.14653/supp-3 Table S2 Supplementary Table 2

## Additional Information and Declarations

### Data Availability

The following information was supplied regarding data availability: The data is available at the China National GeneBank DataBase (CNGBdb): CNP0003559. https://db.cngb.org/search/?q=CNP0003559.

## References

[ref-1] Alexandrov LB, Kim J, Haradhvala NJ, Huang MN, Tian Ng AW, Wu Y, Boot A, Covington KR, Gordenin DA, Bergstrom EN, Islam SMA, Lopez-Bigas N, Klimczak LJ, McPherson JR, Morganella S, Sabarinathan R, Wheeler DA, Mustonen V, Getz G, Rozen SG, Stratton MR, Group PMSW and Consortium P (2020). The repertoire of mutational signatures in human cancer. Nature.

[ref-2] Anglesio MS, Carey MS, Kobel M, Mackay H, Huntsman DG, Vancouver Ovarian Clear Cell Symposium S (2011). Clear cell carcinoma of the ovary: a report from the first Ovarian Clear Cell Symposium, June 24th, 2010. Gynecologic Oncology.

[ref-3] Anglesio MS, Papadopoulos N, Ayhan A, Nazeran TM, Noe M, Horlings HM, Lum A, Jones S, Senz J, Seckin T, Ho J, Wu RC, Lac V, Ogawa H, Tessier-Cloutier B, Alhassan R, Wang A, Wang Y, Cohen JD, Wong F, Hasanovic A, Orr N, Zhang M, Popoli M, McMahon W, Wood LD, Mattox A, Allaire C, Segars J, Williams C, Tomasetti C, Boyd N, Kinzler KW, Gilks CB, Diaz L, Wang TL, Vogelstein B, Yong PJ, Huntsman DG, Shih IM (2017). Cancer-associated mutations in endometriosis without cancer. The New England Journal of Medicine.

[ref-4] Baniak N, Fadare O, Kobel M, De Coteau J, Parkash V, Hecht JL, Hanley KZ, Gwin K, Zheng W, Quick CM, Jarboe EA, Liang SX, Kinloch M (2019). Targeted molecular and immunohistochemical analyses of endometrial clear cell carcinoma show that POLE mutations and DNA mismatch repair protein deficiencies are uncommon. The American Journal of Surgical Pathology.

[ref-5] Brown LC, Tucker MD, Sedhom R, Schwartz EB, Zhu J, Kao C, Labriola MK, Gupta RT, Marin D, Wu Y, Gupta S, Zhang T, Harrison MR, George DJ, Alva A, Antonarakis ES, Armstrong AJ (2021). LRP1B mutations are associated with favorable outcomes to immune checkpoint inhibitors across multiple cancer types. The Journal for ImmunoTherapy of Cancer.

[ref-6] Chandler RL, Damrauer JS, Raab JR, Schisler JC, Wilkerson MD, Didion JP, Starmer J, Serber D, Yee D, Xiong J, Darr DB, Pardo-Manuel de Villena F, Kim WY, Magnuson T (2015). Coexistent ARID1A-PIK3CA mutations promote ovarian clear-cell tumorigenesis through pro-tumorigenic inflammatory cytokine signalling. Nature Communications.

[ref-7] Cisowski J, Bergo MO (2017). What makes oncogenes mutually exclusive?. Small GTPases.

[ref-8] Crotzer DR, Sun CC, Coleman RL, Wolf JK, Levenback CF, Gershenson DM (2007). Lack of effective systemic therapy for recurrent clear cell carcinoma of the ovary. Gynecologic Oncology.

[ref-9] Dionisio de Sousa IJ, Cunha AI, Saraiva IA, Portugal RV, Gimba ERP, Guimaraes M, Prazeres H, Lopes JM, Soares P, Lima RT (2021). LRP1B expression as a putative predictor of response to pegylated liposomal doxorubicin treatment in ovarian cancer. Pathobiology.

[ref-10] DeLair DF, Burke KA, Selenica P, Lim RS, Scott SN, Middha S, Mohanty AS, Cheng DT, Berger MF, Soslow RA, Weigelt B (2017). The genetic landscape of endometrial clear cell carcinomas. Journal of Pathology.

[ref-11] Gadducci A, Lanfredini N, Tana R (2014). Novel insights on the malignant transformation of endometriosis into ovarian carcinoma. Gynecological Endocrinology.

[ref-12] Gerlinger M, Rowan AJ, Horswell S, Math M, Larkin J, Endesfelder D, Gronroos E, Martinez P, Matthews N, Stewart A, Tarpey P, Varela I, Phillimore B, Begum S, McDonald NQ, Butler A, Jones D, Raine K, Latimer C, Santos CR, Nohadani M, Eklund AC, Spencer-Dene B, Clark G, Pickering L, Stamp G, Gore M, Szallasi Z, Downward J, Futreal PA, Swanton C (2012). Intratumor heterogeneity and branched evolution revealed by multiregion sequencing. The New England Journal of Medicine.

[ref-13] Hu H, Yin S, Ma R, Chen R, Li S, Chen Y, Fei H, Yang L (2021). CREBBP knockdown suppressed proliferation and promoted chemo-sensitivity via PERK-mediated unfolded protein response in ovarian cancer. Journal of Cancer.

[ref-14] Hubschmann D, Jopp-Saile L, Andresen C, Kramer S, Gu Z, Heilig CE, Kreutzfeldt S, Teleanu V, Frohling S, Eils R, Schlesner M (2021). Analysis of mutational signatures with yet another package for signature analysis. Genes Chromosomes Cancer.

[ref-15] Jenison EL, Montag AG, Griffiths CT, Welch WR, Lavin PT, Greer J, Knapp RC (1989). Clear cell adenocarcinoma of the ovary: a clinical analysis and comparison with serous carcinoma. Gynecologic Oncology.

[ref-16] Ju B, Wang J, Yang B, Sun L, Guo Y, Hao Q, Wu J (2018). Morphologic and immunohistochemical study of clear cell carcinoma of the uterine endometrium and cervix in comparison to ovarian clear cell carcinoma. The International Journal of Gynecological Pathology.

[ref-17] Jung US, Min KW, Kim DH, Kwon MJ, Park H, Jang HS (2021). Suppression of ARID1A associated with decreased CD8 T cells improves cell survival of ovarian clear cell carcinoma. Journal of Gynecologic Oncology.

[ref-18] Katagiri A, Nakayama K, Rahman MT, Rahman M, Katagiri H, Nakayama N, Ishikawa M, Ishibashi T, Iida K, Kobayashi H, Otsuki Y, Nakayama S, Miyazaki K (2012). Loss of ARID1A expression is related to shorter progression-free survival and chemoresistance in ovarian clear cell carcinoma. Modern Pathology.

[ref-19] Kato N, Sato Y, Kamataki A, Fukase M, Uchigasaki S, Kurose A (2019). PIK3CA hotspot mutations and cyclooxygenase-2 expression in ovarian clear cell carcinomas: a close association with stromal features. Human Pathology.

[ref-20] Kim SI, Lee JW, Lee M, Kim HS, Chung HH, Kim JW, Park NH, Song YS, Seo JS (2018). Genomic landscape of ovarian clear cell carcinoma via whole exome sequencing. Gynecologic Oncology.

[ref-21] Kroeger Jr PT, Drapkin R (2017). Pathogenesis and heterogeneity of ovarian cancer. Current Opinion in Obstetrics and Gynecology.

[ref-22] Kuo KT, Mao TL, Jones S, Veras E, Ayhan A, Wang TL, Glas R, Slamon D, Velculescu VE, Kuman RJ, Shih Ie M (2009). Frequent activating mutations of PIK3CA in ovarian clear cell carcinoma. The American Journal of Pathology.

[ref-23] Li J, Lupat R, Amarasinghe KC, Thompson ER, Doyle MA, Ryland GL, Tothill RW, Halgamuge SK, Campbell IG, Gorringe KL (2012). CONTRA: copy number analysis for targeted resequencing. Bioinformatics.

[ref-24] Lim D, Ip PP, Cheung AN, Kiyokawa T, Oliva E (2015). Immunohistochemical comparison of ovarian and uterine endometrioid carcinoma, endometrioid carcinoma with clear cell change, and clear cell carcinoma. The American Journal of Surgical Pathology.

[ref-25] Machida H, Matsuo K, Yamagami W, Ebina Y, Kobayashi Y, Tabata T, Kanauchi M, Nagase S, Enomoto T, Mikami M (2019). Trends and characteristics of epithelial ovarian cancer in Japan between 2002 and 2015: a JSGO-JSOG joint study. Gynecologic Oncology.

[ref-26] Maru Y, Tanaka N, Ohira M, Itami M, Hippo Y, Nagase H (2017). Identification of novel mutations in Japanese ovarian clear cell carcinoma patients using optimized targeted NGS for clinical diagnosis. Gynecologic Oncology.

[ref-27] Mayakonda A, Lin DC, Assenov Y, Plass C, Koeffler HP (2018). Maftools: efficient and comprehensive analysis of somatic variants in cancer. Genome Research.

[ref-28] Moyano-Galceran L, Pietila EA, Turunen SP, Corvigno S, Hjerpe E, Bulanova D, Joneborg U, Alkasalias T, Miki Y, Yashiro M, Chernenko A, Jukonen J, Singh M, Dahlstrand H, Carlson JW, Lehti K (2020). Adaptive RSK-EphA2-GPRC5A signaling switch triggers chemotherapy resistance in ovarian cancer. EMBO Molecular Medicine.

[ref-29] Munksgaard PS, Blaakaer J (2012). The association between endometriosis and ovarian cancer: a review of histological, genetic and molecular alterations. Gynecologic Oncology.

[ref-30] Murakami R, Matsumura N, Brown JB, Higasa K, Tsutsumi T, Kamada M, Abou-Taleb H, Hosoe Y, Kitamura S, Yamaguchi K, Abiko K, Hamanishi J, Baba T, Koshiyama M, Okuno Y, Yamada R, Matsuda F, Konishi I, Mandai M (2017). Exome sequencing landscape analysis in ovarian clear cell carcinoma shed light on key chromosomal regions and mutation gene networks. The American Journal of Pathology.

[ref-31] Oliveira D, Schnack TH, Poulsen TS, Christiansen AP, Hogdall CK, Hogdall EV (2021). Genomic sub-classification of ovarian clear cell carcinoma revealed by distinct mutational signatures. Cancers.

[ref-32] Pozzati F, Moro F, Pasciuto T, Gallo C, Ciccarone F, Franchi D, Mancari R, Giunchi S, Timmerman D, Landolfo C, Epstein E, Chiappa V, Fischerova D, Fruscio R, Zannoni GF, Valentin L, Scambia G, Testa AC (2018). Imaging in gynecological disease (14): clinical and ultrasound characteristics of ovarian clear cell carcinoma. Ultrasound in Obstetrics & Gynecology.

[ref-33] Sugiyama T, Kamura T, Kigawa J, Terakawa N, Kikuchi Y, Kita T, Suzuki M, Sato I, Taguchi K (2000). Clinical characteristics of clear cell carcinoma of the ovary: a distinct histologic type with poor prognosis and resistance to platinum-based chemotherapy. Cancer.

[ref-34] Takano M, Tsuda H, Sugiyama T (2012). Clear cell carcinoma of the ovary: is there a role of histology-specific treatment?. Journal of Experimental & Clinical Cancer Research.

[ref-35] Travaglino A, Raffone A, Mascolo M, Guida M, Insabato L, Zannoni GF, Zullo F (2020). Clear cell endometrial carcinoma and the TCGA classification. Histopathology.

[ref-36] Vang R, Levine DA, Soslow RA, Zaloudek C, Shih Ie M, Kurman RJ (2016). Molecular alterations of TP53 are a defining feature of ovarian high-grade serous carcinoma: a rereview of cases lacking TP53 mutations in the cancer genome atlas ovarian study. International Journal of Gynecological Pathology.

[ref-37] Wang D, Xu Y, Goldstein JB, Ye K, Hu X, Xiao L, Li L, Chang L, Guan Y, Long G, He Q, Yi X, Zhang J, Wang Z, Xia X, Zhou L (2020). Preoperative evaluation of microvascular invasion with circulating tumour DNA in operable hepatocellular carcinoma. Liver International.

[ref-38] Wiegand KC, Shah SP, Al-Agha OM, Zhao Y, Tse K, Zeng T, Senz J, McConechy MK, Anglesio MS, Kalloger SE, Yang W, Heravi-Moussavi A, Giuliany R, Chow C, Fee J, Zayed A, Prentice L, Melnyk N, Turashvili G, Delaney AD, Madore J, Yip S, McPherson AW, Ha G, Bell L, Fereday S, Tam A, Galletta L, Tonin PN, Provencher D, Miller D, Jones SJ, Moore RA, Morin GB, Oloumi A, Boyd N, Aparicio SA, Shih Ie M, Mes-Masson AM, Bowtell DD, Hirst M, Gilks B, Marra MA, Huntsman DG (2010). ARID1A mutations in endometriosis-associated ovarian carcinomas. The New England Journal of Medicine.

[ref-39] WHO Classification of Tumours Editorial Board (2020). WHO classification of tumours. Female genital tumours.

[ref-40] Yamamoto S, Tsuda H, Takano M, Tamai S, Matsubara O (2012). Loss of ARID1A protein expression occurs as an early event in ovarian clear-cell carcinoma development and frequently coexists with PIK3CA mutations. Modern Pathology.

