# Supplementary figures and images for: Next-generation sequencing shows the genomic features of ovarian clear cell cancer and compares the genetic architectures of high-grade serous ovarian cancer and clear cell carcinoma in ovarian and endometrial tissues
Source: PeerJ. 2023 Jan 26;11:e14653. doi: 10.7717/peerj.14653 (PMC9884475; doi:10.7717/peerj.14653)

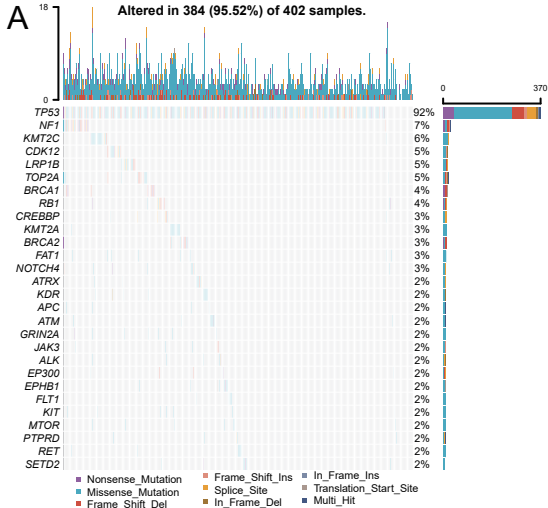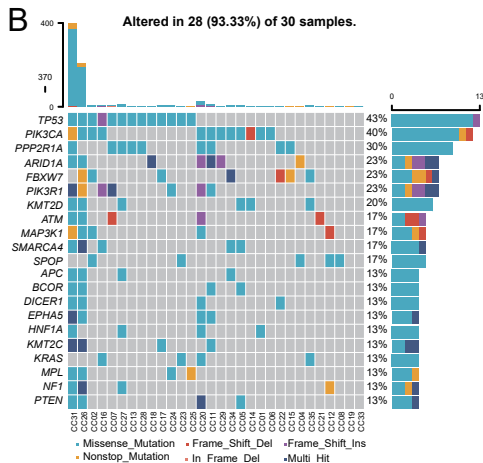

Supplement: Figure S1 — (A) Mutations with high mutational frequencies in HGSOC. (B) Mutations with high mutational frequencies in HGSOC. ECCC, endometrial clear cell carcinoma; HGSOC, high-grade serous ovarian carcinoma. [file peerj-11-14653-s001.pdf]
